# Phytochemicals: Alternative for Infertility Treatment and Associated Conditions

**DOI:** 10.1155/2023/1327562

**Published:** 2023-05-11

**Authors:** Saziini H. Chorosho, Neha Malik, Gulsheen Panesar, Pratima Kumari, Sarita Jangra, Rupinder Kaur, Mariam S. Al-Ghamdi, Tasahil S. Albishi, Hitesh Chopra, Ravinder Singh, H. C. Ananda Murthy

**Affiliations:** ^1^Chitkara College of Pharmacy, Chitkara University, Punjab, India; ^2^Department of Biology, College of Applied Sciences, Umm Al-Qura University, Saudi Arabia; ^3^Department of Applied Chemistry, School of Applied Natural Science, Adama Science and Technology University, P.O. Box 1d8, Adama, Ethiopia; ^4^Department of Prosthodontics, Saveetha Dental College & Hospital, Saveetha Institute of Medical and Technical Sciences (SIMAT), Saveetha University, Chennai, 600077 Tamil Nadu, India

## Abstract

Infertility and obstetric complications have become global health issues in the past few years. Infertility is defined as the inability of a couple to conceive even after twelve months or more of regular and unprotected intercourse. According to WHO data published in the year 2020, 186 million people have infertility globally. Factors leading to infertility are variable in both males and females. But some common factors include smoking, alcohol consumption, obesity, and stress. Various synthetic drugs and treatment options are available that are effective in treating infertility, but their prolonged usage produces various unwanted adverse effects like hot flashes, mood swings, headaches, and weight gain. In extreme cases, these may also lead to the development of anxiety and depression. Herbal remedies have gained a lot of popularity over the years, and people's inclination toward them has increased all over the world. The prime reason is that these show significant therapeutic efficacy and have fewer side effects. The therapeutic efficacy of plants can be attributed to the presence of diverse phytochemical classes of constituents like alkaloids, flavonoids, and volatile oils. These secondary metabolites, or phytomolecules, can be used to develop herbal formulations. The review highlights the applications and mechanisms of action of various phytochemicals for treating infertility. Also, it focuses on the various future prospects associated with it.

## 1. Introduction

**Figure 1:**
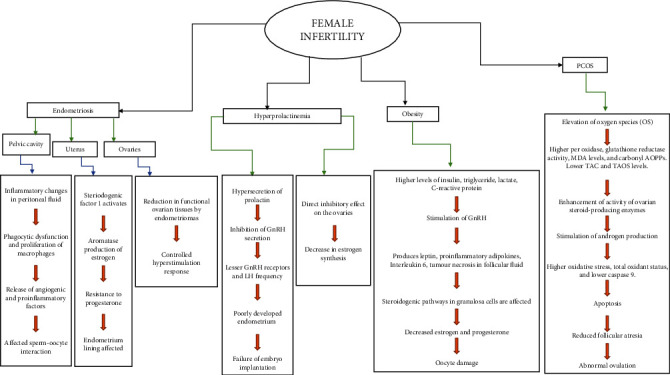
Schematic flow chart of the factors leading to female infertility [110, 111].

According to the WHO, infertility is considered a global health concern, affecting millions of people of reproductive age. According to epidemiological studies, approximately 48 million couples and 186 million individuals worldwide are infertile. Infertility can be defined as a condition of the male or female procreative system characterized by the failure to conceive after 12 months of consistent unprotected sexual contact [1]. In males, it can be due to injury of the testicles, varicocele, testicular cancer, hypogonadism, azoospermia, undescended testicles, and premature ejaculation [2, 3]. Additionally, other factors contributing to male infertility include cystic fibrosis, turner syndrome, and fragile X syndrome [2, 4, 5]. In the case of females, the most common risk factors associated with infertility include a history of tubal pregnancy, abnormal menstruation, ovarian disorders, uterine fibroids, intrauterine adhesion, endometriosis, and uterine polyps. It is also observed that women older than 35 experience problems conceiving; thus, an evaluation of infertility can begin after 6 months, while an immediate evaluation is advised if the woman is over 40. Metabolic disorders like polycystic ovary syndrome (PCOS), diabetes mellitus, and obesity also enhance the epidemiology of infertility in affected couples [6, 7]. The schematic flow chart of the factors leading to female infertility is presented in Figure 1.

**Figure 2:**
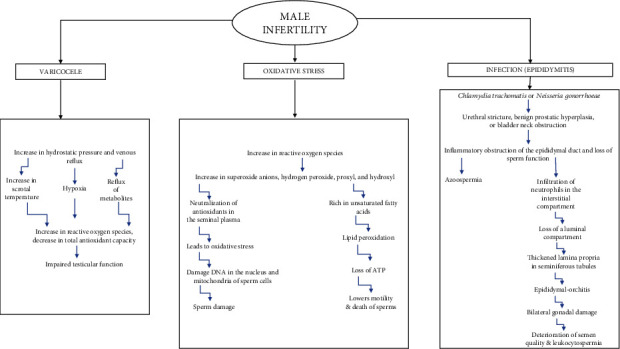
Schematic flow chart of the factors leading to male infertility [112–114].

Furthermore, alcohol consumption, smoking, and toxic environmental exposure like lead and pesticides may enhance the risk of infertility in both sexes [2, 5, 8].  Figure 2 displays the schematic flow chart of the factors leading to male infertility. Over the years, various techniques have been developed through which infertility can be treated in both males and females. For instance, assisted reproductive techniques (ART), intracytoplasmic sperm injection (ICSI), varicocelectomy, and transurethral ejaculatory duct resection (TEDR) are widely used for treating infertility in males, and for females, there are preliminary tubal surgeries, gamete intrafallopian transfer (GIFT), fimbrioplasty, and tubal reanastomosis. Also, intrauterine insemination (IUI) and in vitro fertilization (IVF) are trending treatment options for both female and male infertility [2, 9, 10]. Although these therapies are conventionally used, there are certain limitations too. For example, assisted reproductive techniques (ART) somehow do not assure pregnancy as well as a live birth. Also, the success rate is significantly lower than the failure rate. The mentioned treatments besides being highly expensive and of long duration also impact the mental health of the patients. Infertile couples undergoing such treatments are reported to show high levels of anxiety as well as depression. Literature shows that infertile females face more stress than infertile males because of the social burden of childbearing. Though children conceived through IVF are healthy, IVF is associated with a high risk of perinatal and obstetric results. It may also lead to preterm labor and delivery and underweight babies. It is also related to congenital defects and neurological disorders [11, 12]. Plants and phytochemicals have been used for ages for the treatment of infertility. Numerous plants like Ashoka, Shatavari, Dashmool, and Guduchi have been employed in Ayurveda for treating infertility. Plants are known to treat infertility through diverse mechanisms like improving prematured ovarian failure, improving egg and sperm quality, treating PCOD, and balancing the hormones. A case study published by Asmabi and Jithesh in 2022 showed that treating PCOS through Ayurvedic intervention led to regular ovulation which further led to a healthy pregnancy. Prior to the Ayurvedic intervention, the patient underwent both intrauterine insemination and hormonal therapy for conception, which were unsuccessful. Thus, plants have significant potential to be used for treating infertility and associated conditions. These can be used as such or along with conventional therapies for managing infertility [13, 14]. Besides, the emergent use of plants in treating a number of disease conditions other than infertility has become remarkable. Phytoconstituents such as caflanone present in *Cannabis sativa* L.; crocin extracted from *Crocus sativus* L.; quinadoline B found in *Cladosporium* sp.; delphinidin 3,3′-di-glucoside-5-(6-p-coumarylglucoside) (DGCG) isolated from *Gentiana* cv. Albireo; anethole present in *Foeniculum vulgare* Mill., *Croton grewioides* Baill., etc.; curcumin found in *Curcuma longa* L.; and adlumidine present in *Fumaria indica* have proven effective in tackling the recent COVID-19 outbreak [15]. The recent trends of the application of phyto-nanotechnology in treating neurological disorders (ND) such as Alzheimer's disease (AD) and Parkinson's disease (PD) have proven their significance. The various phytoconstituents incorporated in this application are *α*- and *β*-asarone found in *Acorus calamus*; S-allyl cysteine (SAC) found in *Allium sativum*; curcumin found in *Curcuma longa*; quercetin 3-glucuronide, glycitin, caffeic acid, and protocatechinic acid found in *Coriandrum sativum* L.; hyperoside found in *Hypericum perforatum*; etc. [16]. A natural polyphenolic compound, resveratrol, has gained its popularity in treating not only infertility but also a number of disease conditions by acting as an antioxidant, anticancer, cardioprotective, and anti-inflammatory [17]. Another natural phenolic compound, curcumin, found in the *Curcuma* genus can be formulated for effective drug delivery. It has been useful in treating skin disorders, cancers, neurological disorders, inflammation, multiple myeloma, etc. [18]. This article emphasizes the use of phytochemicals for the treatment of infertility and associated conditions.  Table 1 provides a summary of different herbal plants and the parts of those plants that are used to treat infertility and other related conditions.

## 2. Mechanism of Action of Selected Plant Constituents for Treating Infertility and Associated Conditions

### 2.1. Resveratrol's Role in the Anti-Inflammatory Process in Endometriosis

**Figure 3:**
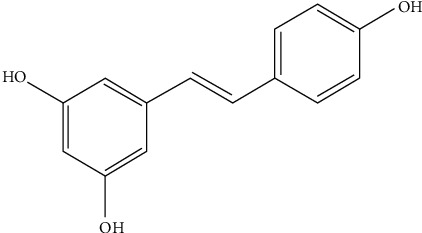
Chemical structure of resveratrol.

Resveratrol (trans-3,5,40-trihydroxystilbene) is a naturally occurring polyphenolic compound found in berries, wine, peanuts, grapes, soy, etc. It is also found in plants such as *Veratrum grandiflorum* and *Polygonum cuspidatum*. It possesses antiatherogenic, antiproliferative, antineoplastic, anti-inflammatory, antiangiogenic, and antineoplastic properties. This compound (Figure 3) works by reducing the cases of endometriosis in females by inhibiting prostaglandin synthesis, a significant anti-inflammatory effect of resveratrol.

Battaglia et al. in 2022 showed that resveratrol enhances the biogenesis in granulosa cells, which in turn improves folliculogenesis. This was done by modifying the miRNome which acts on miRNA involved in the mitochondrial pathway. This led to the quality enhancement of oocytes resulting in better chances of pregnancy [92].

A different study conducted by Illiano et al. in 2020 showed that resveratrol supplements enhanced the concentration of sperm as well as their motility in idiopathic infertility of males [93]. Resveratrol is known to improve the ovarian function by promoting the expression of sirtuin (SIRT) 1 expression in the ovaries. Upregulation of SIRT 1 expression reduces the oxidative stress leading to enhanced telomerase activity which is associated with better ovarian function. Resveratrol also inhibits the aging of endometrial cells which is important for the implantation of an embryo. This is because resveratrol inhibits the expression of CRABP 2-RAR [94]. Thus, resveratrol serves as a potential therapeutic molecule for the management of infertility as well as other disorders associated to it.

Rudzitis-Auth et al., in 2013, experimented on twenty female mice and surgically induced endometriosis by transplanting uterine tissue into the abdominal cavity. This resulted in mesenteric and peritoneal endometriotic lesions. Resveratrol, when administered with a dose of 40 mg/kg/day by oral gavage for a duration of 4 weeks, resulted in the inhibition of angiogenesis in mesenteric and peritoneal endometriotic lesions [95].

Resveratrol exerts varying effects on a number of molecular pathways mainly involved in inflammation, namely, the Ah (aryl hydrocarbon) receptor, Nf-*ĸ*B (nuclear factor kappa-light-chain-enhancer of activated B cells), arachidonic acid, or AP-1 (activator protein-1).

#### 2.1.1. Arachidonic Acid Pathway

Resveratrol interacts with COX-2 (cyclooxygenase-2) in this pathway by suppressing its effects at various stages. A study revealed that resveratrol in the epithelial cells of mammary glands suppresses PMA- (phorbol 12-myristate 13-acetate-) induced cyclooxygenase transcription by mainly inhibiting the protein kinase C pathway and eventually inhibiting inflammation. Moreover, resveratrol also works by preventing the induction of COX-2 (cyclooxygenase-2) promoter activity, which is mediated by c-Jun and ERK-1 (extracellular signal-regulated kinase-1).

#### 2.1.2. Aryl Hydrocarbon Receptor (AhR)

This receptor plays a key role in the immune system as it is a mediator of dioxin toxicity, and dioxin induces immunosuppression. A study concluded that AhR (aryl hydrocarbon receptor) binds to different factors such as Nf-*ĸ*B (nuclear factor kappa-light-chain-enhancer of activated B cells), estrogen receptors, and E2F1. Resveratrol has been identified as producing antagonistic effects on AhR (aryl hydrocarbon receptor). The immune system is regulated by the effector Th17 (T helper 17) subset and FoxP3+ (forkhead box P3) Tregs (regulatory T cells). Different studies revealed that resveratrol could inhibit Th17 (T helper 17) cell and FoxP3+ (forkhead box P3) Treg (regulatory T cell) development. Researchers concluded that resveratrol has a positive outcome in controlling inflammation by inhibiting Th17 (T helper 17) cells.

#### 2.1.3. Activator Protein-1 Pathway

Activator protein-1, together with NFAT (nuclear factor of activated T cells), STATs (signal transducer and activator of transcription proteins), and Nf-*ĸ*B (nuclear factor kappa-light-chain-enhancer of activated B cells), is involved in initiating the inflammatory process. This inflammatory process involves enhancing the transcription of several biomolecules as well as proinflammatory cytokines. AP-1 (activator protein-1) activation is induced by multiple cytokines, mainly by MAPK (mitogen-activated protein kinase) and JNK (c-Jun N-terminal kinase) signaling, cellular stress, growth factors, and hormones. AP-1 (activator protein-1) proteins, when activated, have a major function in T cell differentiation, which causes various inflammatory conditions. Different studies also suggested that this compound inhibits COX-2 (cyclooxygenase-2) activity indirectly by primarily inhibiting the AP-1 (activator protein-1) pathways, interfering with the inflammatory process.

#### 2.1.4. Nf-*ĸ*B (Nuclear Factor Kappa-Light-Chain-Enhancer of Activated B Cells) Pathway

The Nf-*ĸ*B (nuclear factor kappa-light-chain-enhancer of activated B cells) proteins are activated by the phosphorylation of I*ĸ*B (inhibitor of *ĸ*B) proteins. When activated, it activates several target genes, which are responsible for inflammatory responses and cell proliferation. A study suggested that resveratrol suppresses Nf-*ĸ*B (nuclear factor kappa-light-chain-enhancer of activated B cells) activation by blocking TNF-*α* (tumor necrosis factor-alpha) and inducing the activation of Nf-*ĸ*B (nuclear factor kappa-light-chain-enhancer of activated B cells). Different researchers also proved that resveratrol prevents the Nf-*ĸ*B (nuclear factor kappa-light-chain-enhancer of activated B cells) activation through various stimuli other than TNF-*α* (tumor necrosis factor-alpha) like okadaic acid, H_2_O_2_ (hydrogen peroxide), and other proinflammatory cytokines, for instances, LPS (lipopolysaccharide) or IL-1*β* (interleukin-1 beta) [96].

### 2.2. Phytoestrogens in Male Reproductive System

These are naturally occurring nonsteroidal compounds present in many medicinal plants such as *Nigella sativa*, *Punica granatum*, and *Glycyrrhiza glabra*. Phytoestrogens include various categories, namely, lignans, still beans, flavones, and coumestans. These have become popular as abundant literature is available regarding their adverse effects, and also, people are shifting toward plant-derived proteins (soya) which are rich in phytoestrogens [97]. They bind to ER*α* (estrogen receptor alpha) and ER*β* (estrogen receptor beta) by mimicking estradiol's conformational structure. They act on the male reproductive system by interacting with the estrogen receptors and by another mechanism, behaving as an antioxidant or tyrosine kinase inhibitor. It also interferes with the androgen receptor pathway and affects spermatogenesis in males [98].

Cooper and Amber in 2019 reported that phytoestrogens such as soy isoflavones exhibit a similar chemical structure to 17*β*-estradiol. The two major soy isoflavones, namely, daidzein and genistein, bind to estrogen receptor beta (ER*β*), suggesting beneficial effects on fertility, although they are weak estrogens in comparison to endogenous E_2_. The soy isoflavones act by two pathways, namely, the hormonal and nonhormonal pathways, that involve the arrest or alteration of cellular growth through tyrosine kinase or epigenetics [99].

**Figure 4:**
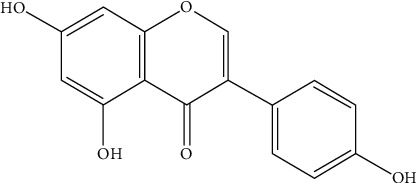
Chemical structure of genistein.

A different study by Desmawati and Sulastri in 2019 revealed the importance of phytoestrogen and genistein (Figure 4) on female fertility. It acts by stimulating progesterone hormone stimulation in the ovaries, estradiol, and cAMP production, maturation of oocytes, and development of zygote in the preimplantation stage of women [100].

### 2.3. Eugenol on Stress-Induced Female Reproductive Dysfunction

**Figure 5:**
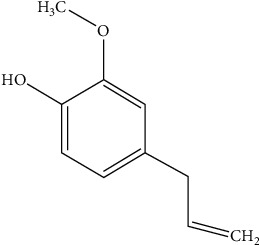
Chemical structure of eugenol.

Eugenol (Figure 5) is a natural phenolic compound (1-allyl-4-hydroxy-3-methoxybenzene) widely present in medicinal plants, namely, *Cinnamomum verum* (cinnamon), *Myristica fragrans* (nutmeg), and *Ocimum basilicum* (basil). It possesses antioxidant, antipyretic, anti-inflammatory, and analgesic properties. In addition, it also has antidepressant properties.

Nikbin et al. in 2020, in their study, showed that eugenol supplements when given with exercise training work by improving the destructive effects of CPF (chlorpyrifos) on testicular tissue at the cellular level, by enhancing PLZF (promyelocytic leukemia zinc finger) and IGF*α* (insulin-like growth factor *α*). This improves the anatomy of the testis, thereby increasing spermatogenesis in males [101].

Stress is a major concern in the present society, affecting all age groups. Females are found to be more susceptible to reproductive disorders related to stress in comparison to males. Chronic stress has a huge impact on sexual dysfunction. It has also shown negative outcomes for subjects who are on infertility treatment. Changes in the reproductive hormonal levels in females have been reported, leading to ovulation disorders and infertility.

In CUMS (chronic unpredictable mild stress), there is a significant elevation of serum cortisol. It is regarded as a major index for triggering stress responses in humans. Chronic stress results in the activation of the HPA (hypothalamic-pituitary-adrenal) axis due to the induction of the hypothalamic release of CRH (corticotropin-releasing hormone), further activating CRHR-1 (corticotropin-releasing hormone receptor-1). Activation of CRHR-1 (corticotropin-releasing hormone receptor-1) stimulates and releases ACTH (adrenocorticotropic hormone) by acting on the adrenal cortex. It further stimulates and produces the hormone cortisol. At a certain level, cortisol helps in the regulation of metabolism and self-maintenance by coping with stressors. But, in constant and prolonged stressful situations, the negative outcome of the HPA (hypothalamic-pituitary-adrenal) axis is impaired. This further leads to the continuous release of serum cortisol. Elevated serum cortisol levels have negative effects such as immune system suppression, cognitive dysfunction, metabolic disturbances, and reproductive functions. In females, the CRHR (corticotropin-releasing hormone receptor) is present in the ovaries and uterus; therefore, it has a role in regulating follicular development and ovarian functions. An elevation in the genetic expression of CRHR-1 (corticotropin-releasing hormone receptor-1) is observed in the ovarian tissues of females induced by stress. In females under prolonged stress, the exaggerated CRH (corticotropin-releasing hormone) effects could further cause inhibition of ovarian steroidogenesis, ovarian failure, and follicular atresia. Eugenol has been shown in studies to interfere with the stress-activated hypothalamic-pituitary-adrenal axis, resulting in a decrease in serum cortisol. Moreover, the HPA (hypothalamic-pituitary-adrenal) axis deactivation concludes the decline of stress-associated overexpression of CRHR-1 (corticotropin-releasing hormone receptor-1). Hence, it can be concluded that eugenol administration could have a positive impact on females struggling with stress-induced reproductive disorders, thereby affecting fertility [102].

### 2.4. Effect of Apigenin on Polycystic Ovary Syndrome

**Figure 6:**
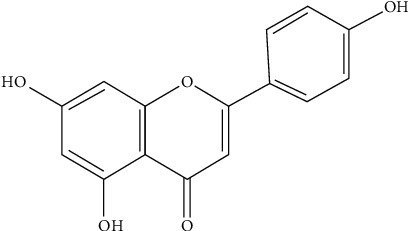
Chemical structure of apigenin.

Apigenin is an active flavonoid that is found in dietary products like parsley, oranges, chamomile tea, celery, or wheat. Chemically, apigenin is referred to as 4′, 5, and 7-trihydroxy flavone. Its chemical formula is C_15_H_10_O_5_ (Figure 6).

In PCOS (polycystic ovary syndrome), a decrease in the number of antral follicles, preantral, granulosa layer thickness, and corpora lutea and an enlargement in theca layer thickness and cystic follicles occur. These changes are caused by an increase in oxidative stress and thus a lack of ovulation. Apigenin significantly inhibits oxidative stress as well as decreases the levels of estradiol and thus significantly enhances the count of corpora lutea and primary and graafian follicles and reduces the count of atretic and cystic follicles. Also, it reduces the theca thickness and increases the thickening of the granulosa layer, thus helping in follicular and ovulation development as there is an increase in normal corpora lutea and normal follicles [103].

Peng et al. experimented on female Sprague-Dawley rats and injected them subcutaneously with DHEA (dehydroepiandrosterone) at a dose concentration of 60 mg/kg for 20 days to induce PCOS (polycystic ovary syndrome). This resulted in a rise in ovarian diameter, body weight, and cysts in the rats. Apigenin with a dose concentration of 20 mg/kg was given by oral gavage to treat PCOS (polycystic ovary syndrome). This resulted in an improved antioxidant status and lipid profile. Also, body weight, ovarian diameter, and cysts were normalized, and healthy follicles were restored [104].

A different study by Park et al. in 2017 reported the antiproliferative as well as apoptotic effects of a plant-derived flavonoid, apigenin, on endometriosis cell lines VK2/E6E7 and End1/E6E7. Apigenin reduces DNA replication and induces cell cycle arrest as well as DNA fragmentation eventually resulting in apoptosis in both the endometriosis cell lines. This process is mediated via the mitochondrial-dependent pathways through the induction of depolarization of ROS (reactive oxygen species) generation, calcium efflux, MMP (mitochondrial membrane potential), and proapoptotic proteins. It also works by inducing ER (endoplasmic reticulum) stress by activating UPR (unfolded protein response) genes. Their apoptotic effects were mediated through the AKT and MAPK (mitogen-activated protein kinase) signal transduction pathways. Hence, apigenin could be considered a novel therapeutic agent in treating endometriosis by activating the integrated intracellular signaling pathways [105].

### 2.5. Effect of Protodioscin in Male Infertility and Impotence

**Figure 7:**
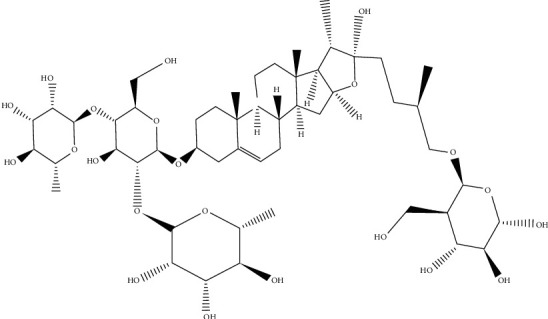
Chemical structure of protodioscin.

Protodioscin is an active compound that is extracted from the leaves of the plant *Tribulus terrestris*. This plant belongs to the family Zygophyllaceae. This compound is categorized as furostanol saponins (Figure 7). It is available under the brand name Libilov™. Over the years, this herb is used to treat male infertility as well as impotence in European and Asian countries [106].

Protodioscin works in the body by activating an enzyme, 5-alpha reductase. This enzyme enhances the production of the hormone testosterone and converts the testosterone into the hormone dihydrotestosterone. Testosterone activates the Sertoli as well as the geminal cells, which leads to more sperm production and spermatogenesis. While dihydrotestosterone works in the Sertoli cells by stimulating the formation of ABP (androgen-binding protein), this leads to the formation of the DHT–ABP (dihydrotestosterone–androgen-binding protein) complex, which enhances spermatozoa's ability to mature into fertile sperm. Moreover, DHT (dihydrotestosterone) stimulates muscle development and helps in the RBC (red blood cell) formation. This RBC formation increases the level of haemoglobin and thus regulates the oxygen transport system as well as the circulation of blood, eventually causing libido [107].

Arsyad experimented on males with oligozoospermia. They treated them with tablets of Libilov, an extract of *Tribulus terrestris* containing an active ingredient, protodioscin, with a dose of 3 × 1 to 3 × 2 tablets per day for 14 to 60 days. This resulted in improved quality and concentration of spermatozoa. It also showed an improvement in orgasm, ejaculation, erection, germinal cells, Sertoli cells, and sexual libido. Moreover, there was an enhancement in the efficiency of the conversion of the hormone testosterone to dihydrotestosterone [107].

A different study by Salgado et al. in 2017 outlined that the main phytoconstituent of the *Tribulus* genus, protodioscin, enhances the chances of fertility in the male by acting on the Sertoli cells, in the seminiferous tubule growth. It converts the hormone testosterone into dihydrotestosterone, thereby playing a major role in male attributes. It also increases serum dehydroepiandrostenedione (DHEA) concentrations resulting in an increase in erectile function [58].

### 2.6. Eurycomanone for Enhancing Male Infertility

**Figure 8:**
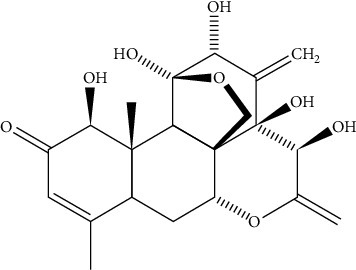
Chemical structure of eurycomanone.

Eurycomanone (Figure 8) is an active quassinoid that is extracted from the roots of *Eurycoma longifolia*, belonging to the family Simaroubaceae. It is commonly called tongkat ali. Chemically, this is written as C_20_H_24_O_9_. This plant is premixed in several beverages and for ages, used in treating male infertility. This quassinoid inhibits two enzymes, phosphodiesterase and aromatase, that are responsible for the synthesis of estradiol.

Due to the reduction of estradiol, there is an increase in the stimulation of the hypothalamus resulting in the secretion of two gonadotropins, the FSH (follicle-stimulating hormone) and the LH (luteinizing hormone). The FSH (follicle-stimulating hormone) and LH (luteinizing hormone) influence the production of testosterone in the Leydig cells. Testosterone along with the FSH (follicle-stimulating hormone) is responsible for regulating spermiogenesis in the seminiferous tubules and thus helps in enhancing fertility in men [108].

In a study by Low et al. in 2013, the major quassinoid, eurycomanone, found in *Eurycoma longifolia* inhibits aromatase conversion of the hormone testosterone into estrogen. Also, at a high dose, it involves in phosphodiesterase inhibition. This leads to an elevation in the level of testosterone steroidogenesis in Leydig cells [109].

## 3. Conclusion

The use of herbal plants for medicinal purposes has been passed down through generations all over the world due to their positive results with minimal side effects and will continue to be used in the future. The main paradigm shift here is that all segments of society cannot afford the costs of synthetic products and surgical treatments. Besides, the success rate is comparatively low, with greater side effects. Moreover, the artificial insemination of hormones is risky and has degraded the quality of life. On the contrary, naturopathic treatment is more cost-effective, has increased bioavailability, has few or no side effects, and has a high success rate. Hence, the switch from synthetic medicines to herbal formulations has been recognized. However, clinical studies and research on medicinal plants should be continued so as to outweigh the use of synthetic products, thereby improving the quality of life. This article comprises various phytochemicals that enhance the fertility rate as well as treat other conditions that are associated with infertility.

## Figures and Tables

**Table 1:** Medicinal plants used for the treatment of infertility and associated conditions.

Sl. no.	Plant	Common/traditional names	Extract/phytoconstituents	Part used	Mode of administration	Dose	Duration	Mechanism of action	References
1	*Cinnamomum zeylanicum* (Lauraceae)	Dalchini	Cinnamon oil (cinnamaldehyde)	Bark	Gavage	100 mg/kg	10 weeks	Increases sperm quality and improves sperm motility, helpful in asthenozoospermic conditions	[14, 19]

2	*Punica granatum* (Lythraceae)	Anaras pomegranate	Pomegranate extract		Gavage	100 mg/kg, 200 mg/kg, 400 mg/kg	8 weeks	Reduces estrogen, andrestandion, and free testosterone hormones in polycystic ovary syndrome (PCOS)	[14, 20–22]
			Pomegranate juice	Fruit	Oral	2 L/week		Increases fertility in male and increases live births	
			Pomegranate juice	Fruit	Oral	50 mg/kg		Reduces testosterone level in polycystic ovary syndrome (PCOS)	
			Methanol extract of pomegranate pericarp (PME)	Peel	Oral	100 mg/kg		Inhibits proliferation of cervical, endometrial, and ovarian carcinoma cell lines	

3	*Withania somnifera* (L.) Dunal (Solanaceae)	Ashwagandha, Indian ginseng	Hydroalcoholic extract (combination of *Withania somnifera* and *Tribulus terrestris*)	Root (WS), fruit (TT)	Oral	100 mg/kg (WS), 98 mg/kg (TT)	28 days	Increases follicle-stimulating hormone (FSH) level	[14, 23, 24]
								Decreases luteinizing hormone (LH) and testosterone levels	
								Prevents ovarian dysfunction	
			Methanolic extract	Roots		400 ng/*μ*L		Reduces preantral and antral follicles and corpus luteum	
								Increases gonadotropin hormone secretion and thus improves the process of oogenesis via GABA- (gamma-aminobutyric acid-) mimetic properties	

4	*Matricaria chamomilla* (chicory)	Chamomile	Hydroalcoholic extract	Whole plant	Oral	25 mg/L, 50 mg/L	12 days	Increases progesterone, dehydroepiandrosterone, and 17*β*-estradiol levels	[14, 25, 26]
								Decreases ROS (reactive oxygen species), antrum formation, and follicular diameter	
								Prolongs oocyte survival	
			Aqueous extract	Flower	Oral	5 mL, twice daily	4 weeks	Decreases serum prolactin levels in idiopathic hyperprolactinemia	

5	*Camellia sinensis* (Theaceae)	Chinese tea	Black tea extract	Leaves	Oral	1 mL/100 g body weight daily	28 days	Increases serum estradiol	[14, 27–29]
								Decreases estrogen-dependent menopausal symptoms	
			Crude phenolic extract	Leaves	Oral	100, 200, and 400 mg/kg body weight	15 days	Increases serum prolactin level	
			Green tea extract	Leaves		50, 100, and 200 mg/kg body weight	29 days	Restores concentration and secretion of FSH (follicle-stimulating hormone), LH (luteinizing hormone), estradiol, and testosterone in letrozole-induced PCOS (polycystic ovary syndrome)	

6	*Phoenix Dactylifera* (Arecaceae)	Date palm	Ethanol extract		Gavage	25, 50, 100, 150, 200, and 250 mg/kg/day	8 weeks	Decreases levels of testosterone and estradiol	[14, 30]

7	*Foeniculum vulgare* (Umbelliferae)	Fennel	Acetone extract	Fruit	Oral	5-10 g	15 days (male)	Decreases total protein concentration in the vas deferens and testes but increases in the prostate gland and seminal vesicles	[14, 31, 32]
			Fennel oil				10 days (female)	Leads to vaginal cornification and oestrus cycle	
								Increases the growth of mammary glands and induces prolactin secretion for the presence of dianethole and photoanethole	

8	*Nigella sativa* (Ranunculaceae)	Black cumin	Hydroalcoholic extract	Seed	Oral	50, 100, and 200 mg/kg	30 days	Increases LH (luteinizing hormone) and testosterone and estrogen levels and decreases progesterone and antioxidant enzymes in PCOS (polycystic ovary syndrome)	[14, 33, 34]
			Ethanol extract	Seed	Parenteral	50 *μ*g/mL	20 days	Increases rate of maturation, fertilization, and blastocyst formation	
								Increases ovulation in PCOS (polycystic ovary syndrome) by reducing LH (luteinizing hormone) dominance over FSH (follicle-stimulating hormone)	

9	*Glycyrrhiza glabra* (Fabaceae)	Licorice—liquorice	Ethanol extract	Plant	Gavage	100-150 mg/kg/day	3 weeks/21 days	Decreases ovarian cyst in PCOS (polycystic ovary syndrome)	[14, 35, 36]
								Improves oocyte fertilization rate and embryonic development in PCOS (polycystic ovary syndrome)	
			Ethanol extract	Root	Oral	3000 mg/kg/day	6 weeks	Reduces endometrial implants in endometriosis by inhibiting COX-2 (cyclooxygenase-2) and IL-6 (interleukin-6) and decreasing expression of VEGF (vascular endothelial growth factor)	

10	*Crataegus monogyna* (Rosaceae)	Hawthorn	Aqueous extract	Fruit	Gavage	20 mg/kg/day BW	28 days	Increases semen quality and decreases DOX (doxorubicin) toxic effects	[37, 38]
								Increases testosterone, LH (luteinizing hormone), and FSH (follicle-stimulating hormone) levels	

11	*Crocus sativus* L. (Iridaceae)	Saffron	Saffron aqueous extract (crocin and safranal)	Stigma	Oral	200 mg, every morning	10 days	Increases erectile function in erectile dysfunction condition and increases sexual desires and intercourse satisfaction	[39–41]
			Hydroalcoholic extract		Parenteral	1, 2, and 4 dg/kg	10 days	Increases the concentration of LH (luteinizing hormone), FSH (follicle-stimulating hormone), and estradiol levels, produces significant effect on ovarian weight, and enhances folliculogenesis, thereby increasing secondary follicles in the ovary	

12	*Nigella sativa* L. (Ranunculaceae)	Black cumin	*Nigella sativa* oil	Seed	Oral	2.5 mL, twice a day	2 months	Improves sperm count, pH, and motility	[39, 42–44]
								Also improves semen quality and volume	
			Hydroalcoholic *N. sativa* seed extract	Seed				Increases corpus luteum and folliculogenesis	
			*N. sativa* capsule		Oral	2 g, per day	3 months	Increases levels of serum LH (luteinizing hormone), FSH (follicle-stimulating hormone), and testosterone, increases semen volume and quality, and also increases sperm count and motility	

13	*Sesamum indicum* L. (Pedaliaceae)	Sesame	Ethanol extract		Oral	100 mg/mL, 300 mg/mL	15 days	Increases FSH (follicle-stimulating hormone)	[39, 45]
								Decreases the total cholesterol levels which are increased in PCOS (polycystic ovary syndrome)	

14	*Trigonella foenum-graecum* L. (Leguminosae)	Fenugreek	Seed extract (Furocyst)	Seed	Oral	500 mg, twice daily	3 months or 90 days	Increases levels of FSH (follicle-stimulating hormone) and LH (luteinizing hormone)	[46, 47]
								Decreases ovarian cysts and ovarian volume in PCOS (polycystic ovary syndrome)	
								Causes regularity in menstruation	

15	*Lavandula angustifolia* (Lamiaceae)	Lavender	Lavandula essential oil	Aerial parts	Parenteral	5 mg/kg, 10 mg/kg, and 20 mg/kg	14 days	Improves serum FSH (follicle-stimulating hormone) levels	[48, 49]
								Improves sperm viability and motility	

16	*Petroselinum sativum* Hoffm. (Apiaceae)	Parsley	Hydroethanolic extract	Aerial parts	Oral	500 and 1000 mg/kg	28 days	Increases uterine protein levels and serum estradiol	[48, 50]
			Polyphenolic extract			200 mg/kg	28 days	Produces protective effects on fallopian tubes as inflammation or infection may cause infertility in females	

17	*Fumaria parviflora* Lam. (Fumariaceae)	Indian fumitory	Ethanolic extract	Leaves	Gavage, once daily	100, 200, and 400 mg/kg body weight	70 days	Increases sperm count, serum testosterone level, sperm density, number of Leydig cells, spermatocytes, spermatozoids, spermatogonium, and weight of the testis and epididymis	[51]

18	*Allium sativum* (Amaryllidaceae)	Garlic	Aqueous garlic extract		Gavage(gastro-oral), once daily	60 or 120 mg/kg	28 days	Decreases the semen MDA (malondialdehyde) activity, weakens the chromic chloride effects on semen TAS (total antioxidant status), and enhances male fertility due to its antioxidant properties. These mechanisms suggest the protection of semen oxidation, a major cause of male infertility.	[51–53]

19	*Origanum vulgare* (Lamiaceae)	Oregano	Essential oil obtained by hydrodistillation	Leaves	Incubation	1.5 *μ*L	5-10 minutes	Improves the parameters of mobility of sperms such as VCL (curvilinear velocity), VAP (average path velocity), ALH (amplitude of lateral head displacement), and VSL (linear velocity)	[53–55]
			Ethanol extract	Leaves	Intraperitoneal	100-400 mg/kg	28 days	Increases LH (luteinizing hormone) and testosterone levels eventually increasing sperm density	

20	*Panax ginseng* Meyer (Araliaceae)	Ginseng	Aqueous extract		Oral	200 mg/kg	6 months	Improves germ cell counts, Sertoli cells, sperm number, and Sertoli cell index in aged males	[37, 56]
								Improves spermatogenesis by increasing testicular antioxidant concentration (ascorbic acid, glutathione, and *α*-tocopherol) and upregulated expressions of fatty acid-binding protein 9, triosephosphate isomerase 1 protein, and phosphatidylinositol transfer protein	

21	*Tribulus terrestris* L. (Zygophyllaceae)	Puncture vein	Dried extract (androsten capsule)		Oral	250 mg (contains 37.5 mg protodioscin), every 8 hours	3 months	Increases serum DTH (dihydrotestosterone) concentration and improves sperm count and motility	[57, 58]

22	*Chlorophytum borivilianum* (Liliaceae)	Safed musli	Aqueous extract	Root	Oral by gavage, once daily	125 mg/kg/day and 250 mg/kg/day	28 days	Increases sperm count and enhances sexual arousal and libido	[57, 59, 60]
			Water soluble extract	Root tubers	Oral	500 mg, in two divided doses	12 weeks	Improves the quantity and quality of sperm and serum testosterone level	

23	*Mucuna pruriens* (Fabaceae)	Kapikacchu	Seed powder	Seed	Oral	5 g/day	90 days	Increases concentration of sperm in oligozoospermic males and also increases sperm motility in asthenozoospermic condition	[57, 61]
								Improves semen quality	

24	*Cynomorium coccineum* L. & *Cynomorium songaricum* Rupr. (Cynomoriaceae)	Desert thumb	Water extract (*Cynomorium coccineum*)		Oral	47 mg/100 kg BW	6 days	Increases ovarian weight and folliculogenesis and elicits changes in gonadotrophin levels	[57, 62]
		Suo Yang (Chinese)	*Cynomorium songaricum*		Oral	1 g/kg/day	56 days	Increases epididymal sperm count and testis weight, increases GDNF (glial cell-derived neurotrophic factor) expression, and enhances spermatogenesis	

25	*Butea superba* (Leguminosae)	Red Kwao Krua	Ethanolic extract	Tuberous root	Oral	0.01, 0.1, or 1.0 mg/kg BW, daily	6 months	Increases sperm count and enhances sperm motility	[57, 63, 64]
			Powdered crude	Tuber root	Enteral (gastric tube)	2, 25, 250, and 1250 mg/kg/day	8 weeks	Increases sperm count and weight of the testis	

26	*Anthocleista vogelii* Planch (Loganiaceae)	Cabbage tree	Ethanolic extract	Leaves	Oral	100 and 200 mg/kg BW	14 days	Increases estradiol level and induces production of estrogen	[65]
								Decreases CD4+ (cluster of differentiation 4) and CD8+ (cluster of differentiation 8) cytokine production	
								Increases the activation of monocytes and granulocytes	

27	*Moringa oleifera* (Moringaceae)	Drumstick tree	Ethanolic extract	Leaves		20, 50, and 100 *μ*g/mL	28-29 hours	Improves the rate of oocyte maturation	[66–68]
			Hexane extract	Leaves	Gavage	0.5, 5, and 50 mg/30 g BW daily	21 days	Increases epididymal maturity thus enhancing spermatogenesis	
								Increases the testis, seminal vesicles, and epididymis weights	
								Increases thickness of the epididymal wall and seminiferous tubule diameter	

28	*Prunus persica* (Rosaceae) Peach gum polysaccharides	Peach	Hydroalcoholic extract (ethanol)	Trunk	Gavage, twice daily	100 mg/kg	21 days	Increases sperm motility, sperm density, and normal sperm morphology rate	[69]

29	*Arctium lappa* L. (Compositae)	Burdock	Ethanolic extract	Root	Oral	200 or 300 mg/kg	1 month	Increases serum levels of LH (luteinizing hormone), FSH (follicle-stimulating hormone), and testosterone in nondiabetic condition	[70]
								Also increases sperm count and sperm viability in nondiabetic condition	

30	*Lycium barbarum* (Solanaceae)	Wolfberry	Ethanolic extract	Fruit	Intragastric	20% for 0.2 g/kg, 40% for 0.4 g/kg, and 60% for 0.6 g/kg (once per day)	5 days	Increases sperm motility, density, and rate of normal sperm morphology rate	[69]
								Provides protective effect on male spermatogenesis, induced by cyclophosphamide	

31	*Globularia alypum* L. (Globulariaceae)	Turbith	Aqueous extract	Leaves	Oesophagus cannula	300 mg/kg/day and 600 mg/kg/day	30 days	Activates spermatogenesis in males	[71, 72]
						100 mg/kg/day	15 days		

32	*Zingiber officinale* (Zingiberaceae)	Ginger	Methanolic extract	Root	Oral	50, 100, and 150 mg/kg BW	48 days	Increases sperm count and sperm motility and increases seminiferous testicular volume	[71, 73]
								Increases testosterone level as well	
								Decreases testicular damage, induced by busulfan	

33	*Glycyrrhiza uralensis* Fisch (Leguminosae)	Licorice	Licorice extract	Rhizome		0.2, 2, and 20 *μ*mol/L	72 hours	Increases spermatogonia proliferation and spermatocyte differentiation	[46, 74]

34	Morinda officinalis (Rubiaceae)	Indian mulberry	Morindae radix aqueous extract	Root	Incubation	10, 50, 100, and 250 mg/mL	24 hours	Increases levels of testosterone in H_2_O_2_- (hydrogen peroxide-) induced oxidative stress conditions	[46, 75]

35	*Taraxacum officinale* (Asteraceae)	Dandelion	Aqueous extract			1, 10, 25, and 50 mg/mL	12 and 48 hours	Increases testosterone levels in Leydig cells of males	[46, 76]
								Increases the protein and mRNA (messenger ribonucleic acid) levels of steroidogenic enzymes	

36	*Typha capensis* (Rohrb.) N.E.Br. (Typhaceae)	Bulrush	Aqueous extract	Rhizomes	Incubation	10 and 100 *μ*g/mL	24 and 96 hours	Increases testosterone levels	[46, 77]
			F1 fraction						

37	*Ferula hermonis* Boiss (Apiaceae)	Lebanese viagra	Methanolic extract	Root	Oral	6 mg/kg	4 days	Improves LH (luteinizing hormone), FSH (follicle-stimulating hormone), estrogen, and progesterone levels thus helping in the maturation and growth of immature oocytes	[78]
								It also helps in uterine and endometrial development.	

38	*Justicia insularis* T. Anders (Acanthaceae)		Aqueous extract	Leaves	Oral	50 mg/kg	20 days	Induces folliculogenesis and thus enhances corpus luteum number as well as implantation sites	[79]

39	*Prunus mume* (Rosaceae)	Chinese plum	Methanolic extract (3,4-dihydroxybenzaldehyde)	Fruit	Incubation		4 days	Increases estradiol secretion by granulosa cells via enhancing SF-1 (steroidogenic factor-1) gene expression and thus improves the quality of oocytes	[80]

40	*Ginkgo biloba* Linn. (Ginkgoaceae)	Maidenhair tree	*Ginkgo biloba* extract	Leaves	Gavage	50 mg/kg	One time	Increases testosterone and FSH (follicle-stimulating hormone) levels	[46, 81]
								Increases primary spermatocytes number, Leydig cells, round spermatids, and seminiferous tubule diameter	

41	*Eurycoma longifolia* Jack (Simaroubacea)	Tongkat ali	*Eurycoma longifolia* extract		Oral	8 mg/kg BW	14 days	Increases sperm motility and sperm count, spermatogenesis, and testicular function	[46, 82]
								Decreases estrogen levels in males	

42	*Loranthus micranthus* Linn. (Loranthaceae)		Aqueous methanolic extract	Leaves	Oral	100 and 200 mg/kg BW	14 days	Increases sperm viability and motility, Leydig cell count, testis weight, and diameter of seminiferous tubules	[46, 83]
								Decreases sperm abnormalities	

43	*Pedalium murex* Linn. (Pedaliaceae)	Bada Gokhru	Methanol fruit fraction	Fruit	Oral	50 and 10 mg/kg BW	60 days	Increases fertility in males	[42, 84]
								Increases sperm motility and density, spermatogenesis, spermatocytes, spermatids, and interstitial and germinal cell count	
								Increases LH (luteinizing hormone), FSH (follicle-stimulating hormone), and testosterone levels	

44	*Senecio biafrae* (Oliv. & Hiern) J. Moore (Compositae)		Aqueous extract	Stems and leaves	Oral	8, 32, 64, and 128 mg/kg BW	20 days	Increases progesterone and estradiol levels and increases uterine weight	[85]

45	*Milicia excelsa* (Moraceae)	African teak	Aqueous extract	Root	Oral	14, 77, and 140 mg/kg/day BW	7 and 15 days	Increases LH (luteinizing hormone), FSH (follicle-stimulating hormone), estradiol, and progesterone levels	[46, 86]
								Decreases amenorrhea problems	

46	*Eucalyptus robusta* Smith (Myrtaceae)	Safeda	Methanolic extract	Leaves	Oral gavage, once daily	25 mg/kg BW	9 days	Decreases endometritis in females by decreasing SAA (serum amyloid A), iNOS (inducible nitric oxide synthase), and NO (nitric oxide) and increasing COX-2 (cyclooxygenase-2)	[87]

47	*Cyperus rotundus* L. (Cyperaceae)	Nut grass	Water extract	Tubers	Oral	31.68 mg/kg/day	7 days	Increases LIF (leukemia inhibitory factor) and its binding to receptors in endometrium, thereby increasing integrins such as *α*V*β*3 (alpha-v beta-3) and *α*V*β*5 (alpha-v beta-5), resulting in increased trophoblastic cells adhesion and blastocyst implantation	[88]

48	*Apium graveolens* (Umbelliferae)	Celery	Aqueous extract	Leaves	Oral	100 and 200 mg/kg BW	1 month	Increases seminiferous tubule diameter	[33, 89]
								Increases spermatocytes number, spermatozoids, and spermatogonia	
								Increases the number of spermatids with the intake of 200 mg/kg	
								Increases the weights of the vas deferens, testes, and cauda epididymis	

49	*Phaleria macrocarpa* (Thymelaeacea)	Mahkota dewa	Aqueous extract	Fruit	Oral	240 mg/kg	7 weeks	Increases sperm viability without any changes in sperm motility and morphology	[37, 90]
								Improves sperm quality	

50	*Anacyclus pyrethrum* DC (Asteraceae)	Akarkara	Ethanolic extract	Root	Oral	50, 100, and 150 mg/kg	28 days	Increases sperm count, sperm viability, and sperm motility	[37, 91]
								Increases the testis weight, epididymis, seminal vesicles, and prostate	
								Increases levels of LH (luteinizing hormone), FSH (follicle-stimulating hormone), and testosterone	
								Enhances spermatogenesis	

## Data Availability

The data used to support the findings of this study are included within the article.
